# Impact of nutritional status on prognosis in acute myocardial infarction patients undergoing percutaneous coronary intervention

**DOI:** 10.1186/s12872-021-02448-x

**Published:** 2022-01-07

**Authors:** Daisuke Kanda, Yoshiyuki Ikeda, Takuro Takumi, Akihiro Tokushige, Takeshi Sonoda, Ryo Arikawa, Kazuhiro Anzaki, Ippei Kosedo, Mitsuru Ohishi

**Affiliations:** Department of Cardiovascular Medicine and Hypertension, Graduate School of Medical and Dental Sciences, Kagoshima University, 8-35-1 Sakuragaoka, Kagoshima City, Kagoshima 890-8520 Japan

**Keywords:** Acute myocardial infarction, Malnutrition, GNRI, GRACE risk score

## Abstract

**Background:**

Malnutrition affects the prognosis of cardiovascular disease. Acute myocardial infarction (AMI) has been a major cause of death around the world. Thus, we investigated the impact of malnutrition as defined by Geriatric Nutritional Risk Index (GNRI) on mortality in AMI patients.

**Methods:**

In 268 consecutive AMI patients who underwent percutaneous coronary intervention (PCI), associations between all-cause death and baseline characteristics including malnutrition (GNRI < 92.0) and Global Registry of Acute Coronary Events (GRACE) risk score were assessed.

**Results:**

Thirty-three patients died after PCI. Mortality was higher in the 51 malnourished patients than in the 217 non-malnourished patients, both within 1 month after PCI (p < 0.001) and beyond 1 month after PCI (p = 0.017). Multivariate Cox proportional hazards regression modelling using age, left ventricular ejection fraction and GRACE risk score showed malnutrition correlated significantly with all-cause death within 1 month after PCI (hazard ratio [HR] 7.04; 95% confidence interval [CI] 2.30–21.51; p < 0.001) and beyond 1 month after PCI (HR 3.10; 95% CI 1.70–8.96; p = 0.037). There were no significant differences in area under the receiver-operating characteristic (ROC) curve between GRACE risk score and GNRI for predicting all-cause death within 1 month after PCI (0.90 vs. 0.81; p = 0.074) or beyond 1 month after PCI (0.69 vs. 0.71; p = 0.87). Calibration plots comparing actual and predicted mortality confirmed that GNRI (p = 0.006) was more predictive of outcome than GRACE risk score (p = 0.85) beyond 1 month after PCI. Furthermore, comparison of p-value for interaction of malnutrition and GRACE risk score for all-cause death within 1 month after PCI, beyond 1 month after PCI, and the full follow-up period after PCI were p = 0.62, p = 0.64 and p = 0.38, respectively.

**Conclusions:**

GNRI may have a potential for predicting the mortality in AMI patients especially in beyond 1 month after PCI, separate from GRACE risk score. Assessment of nutritional status may help stratify the risk of AMI mortality.

## Background

Acute myocardial infarction (AMI) is a major cause of death around the world [1], and assessment of the mortality risk in AMI patients is crucial when making medical decisions. The Global Registry of Acute Coronary Events (GRACE) risk score is useful for estimating in-hospital mortality by accounting for 8 factors: age, heart rate, systolic blood pressure, initial serum creatinine level, Killip class, cardiac arrest state on hospital admission, elevated levels of cardiac markers, and ST-segment deviation [2]. This risk score is widely used [3, 4], and guidelines from both the European Society of Cardiology and the Japanese Circulation Society recommend the use of this risk score in estimating AMI patient mortality [5, 6].

Based on the accumulated evidence, frailty and sarcopenia are thought to represent important factors that affect cardiovascular disease prognosis [7]. Frailty is defined as a state of decreased physical reserve that leads to adverse outcomes after a stressor event, and consists of physical, mental, and social factors. Since sarcopenia is the main cause of physical frailty and is influenced by nutritional status, malnutrition has been shown to be the main factor responsible for frailty [8, 9]. The Geriatric Nutritional Risk Index (GNRI) is widely used as a simple method for screening nutritional status using body mass index (BMI) and serum albumin [10]. Recent studies have demonstrated that a low GNRI is associated with worsened prognosis among heart failure patients [11], and with mortality among patients with critical limb ischemia [12]. However, the association between malnutrition and mortality in AMI patients remains unclear. The purpose of this study was to investigate the impact of malnutrition as assessed by GNRI on mortality in AMI patients after primary percutaneous coronary intervention (PCI) separate from GRACE risk score.

## Methods

### Study design and subjects

We evaluated a retrospective cohort in a single centre, comprising 268 consecutive AMI patients who were admitted to Kagoshima University Hospital between January 2015 and March 2019. This study was approved by the Research and Ethics Committee of Kagoshima University Hospital and was performed in accordance with the ethical principles stated in the 1975 Declaration of Helsinki. All patients provided written informed consent.

All patients presented with the chief complaint of chest pain and/or dyspnoea at the time of hospital admission. Patients underwent emergent coronary angiography and successful revascularization of the culprit lesion by primary PCI using second-generation drug-eluting stents. All patients were administered dual antiplatelet therapy (aspirin and a thienopyridine, as either prasugrel or clopidogrel) and intravenous heparin before PCI. Patients were followed-up at our hospital or by their physician. We examined the relationship between malnutrition and death within 1 month after PCI, beyond 1 month after PCI, and for the full follow-up period after PCI, excluding only those patients who could not be tracked after discharge.

### Measurements and assessments of GNRI and GRACE risk score

In this study, laboratory values were obtained prior to PCI. The estimated glomerular filtration rate (eGFR) was calculated using the Modification of Diet in Renal Disease equation with coefficients modified for Japanese populations, as follows: eGFR (ml/min/1.73 m^2^) = 194 × serum creatinine (mg/dL)^−1.094^ × age (years)^−0.287^ (× 0.739 for female subjects) [13]. In addition, echocardiograms including left ventricular ejection fraction (LVEF) were also performed on admission.

This study assessed nutritional status using the GNRI, calculated using the following equation: GNRI = 14.89 × serum albumin level (g/dL) + 41.7 × (body weight in kilograms/ideal body weight in kilograms). Body weight/ideal body weight was set at 1 when the bodyweight of the patient exceeded the ideal bodyweight. Ideal bodyweight was calculated using a BMI of 22 kg/m^2^. BMI was calculated as bodyweight in kilograms divided by height in metres squared. Furthermore, GRACE risk score in this study was calculated to estimate the risk of mortality for individual patients at the time of admission. The GRACE risk score calculation was performed online (www.outcomes.org/grace) using the scores for each individual predictive factor (age, heart rate, systolic blood pressure, initial serum creatinine level, Killip class, cardiac arrest state on hospital admission, elevated cardiac markers, and ST-segment deviation).

### Definitions of clinical characteristics

Patients with GNRI < 92.0 at baseline were defined as the malnourished group based on previously published thresholds [10]. ST-segment elevation myocardial infarction (STEMI) was defined as AMI with persistent ST-segment elevation on the electrocardiogram, while non-STEMI was defined as AMI without persistent ST-segment elevation on the electrocardiogram. Current smokers were defined as those patients who has smoked at least 100 cigarettes in their lifetime and who currently smokes cigarettes at the time of admission. Cardiac death was defined as death from mechanical complications, heart failure, arrhythmia, or unexpected sudden death.

### Statistical analysis

Quantitative data are presented as mean ± standard deviation or median and interquartile range (IQR). Fisher’s exact test was used to compare the incidence of categorical variables, with these categorical variables expressed as percentages. Continuous variables were compared between malnourished and non-malnourished groups using Student’s t-test (for data showing a normal distribution) or the Wilcoxon rank-sum test (for data showing a non-normal distribution). Cumulative survival rates for malnourished patients were estimated using a Kaplan–Meier curve that was evaluated using a log-rank test. Cox proportional hazards regression analysis was used to analyse all-cause death, with the results expressed as the hazard ratio (HR) and 95% confidence interval (CI). Independent associations between all-cause death and baseline characteristics were assessed by multivariate Cox proportional hazards model analysis using relevant factors, defined as variables with p < 0.05 on univariate analysis. In addition, we conducted a test for interaction of malnutrition and GRACE risk score. Receiver-operating characteristic (ROC) curves analyses in order to evaluate the GNRI and GRACE risk scorers for all-cause death after PCI were performed, and compared these area under the curves (AUCs). Furthermore, the goodness of fit of logistic analysis for GRACE risk score and GNRI for predicting all-cause death after PCI were analysed by Hosmer and Lemeshow test. Calibration plots were used to evaluate the validity of the GNRI and GRACE risk score for AMI patients mortality after PCI. Values of p < 0.05 were considered indicative of statistical significance. JMP® 16 (SAS Institute Inc., Cary, NC, USA) and R (version 4.1.1; The R Foundation for Statistical Computing, Vienna, Austria) were used for statistical analysis.

## Results

### Baseline characteristics

Table 1 shows the baseline clinical characteristics of the patients. Fifty-one patients (19%) were malnourished (GNRI < 92.0), while 217 patients (81%) were not. Malnourished patients were older median age, 71 years; IQR, 66–83 years) than non-malnourished patients (median, 67 years; IQR, 57–75 years; p < 0.001). Both eGFR and LVEF were lower in the malnourished group than in the non-malnourished group (p < 0.001 each). In addition, GRACE risk score was significantly higher in the malnourished group (median, 166; IQR, 132–188) than in the non-malnourished group (median, 124; IQR, 104–148; p < 0.001).

**Table 1:** Baseline characteristics of study patients according to nutritional status

Variables	Overall	Malnourished group	Non-malnourished group	P value
	(n = 268)	(n = 51)	(n = 217)	
Age, years	68 [59, 76]	71 [66, 83]	67 [57, 75]	< 0.001
Sex: male, n (%)	186 (69)	29 (59)	157 (72)	0.042
BMI, kg/m^2^	24.0 [21.6, 26.4]	20.7 [18.5, 22.3]	24.5 [22.5, 26.7]	< 0.001
Risk factors, n (%)				
Hypertension	194 (72)	31 (61)	163 (75)	0.055
Diabetes mellitus	148 (55)	30 (59)	118 (54)	0.64
Dyslipidaemia	174 (65)	23 (45)	151 (69)	0.002
Current smoker	60 (22)	4 (8)	56 (25)	0.005
Haemodialysis	35 (13)	12 (24)	23 (11)	0.020
Medication, n (%)				
Aspirin	86 (32)	15 (29)	71 (33)	0.74
Thienopyridines	52 (19)	13 (25)	39 (18)	0.24
Dual antiplatelet therapy	32 (12)	6 (12)	26 (12)	1.00
Oral anticoagulation	16 (6)	8 (16)	8 (4)	0.004
Calcium-channel blocker	92 (34)	16 (31)	76 (35)	0.73
ACEI	13 (5)	4 (8)	9 (4)	0.28
ARB	73 (27)	11 (22)	62 (29)	0.38
β-blocker	40 (15)	7 (14)	33 (15)	1.00
Statin	99 (37)	16 (31)	83 (38)	0.42
Laboratory data				
LDL-C, mg/dL	96 [73, 123]	78 [61, 108]	100 [77, 130]	< 0.001
HDL-C, mg/dL	46 [40,58]	46 [37, 62]	47 [40, 57]	0.78
Triglyceride, mg/dL	99 [73, 146]	72 [49, 93]	109 [80, 158]	< 0.001
Albumin, g/dL	3.8 [3.5, 4.2]	3.0 [2.6, 3.3]	4.0 [3.7, 4.3]	< 0.001
Uric acid, mg/dL	5.6 [4.3, 6.8]	6.2 [5.0, 8.1]	5.5 [4.3, 6.7]	0.022
FPG, mg/dL	129 [107,177]	141 [104, 219]	127 [107, 171]	0.157
Haemoglobin A1c, %	6.3 [5.6, 7.2]	6.5 [5.6, 7.7]	6.3 [5.6, 7.2]	0.60
Creatinine, mg/dL	0.95 [0.75,1.32]	1.36 [0.88, 3.12]	0.91 [0.74,1.19]	< 0.001
eGFR, mL/min/1.73 m^2^	54.7 [37.8, 72.2]	40.6 [13.8, 54.5]	57.1 [41.6, 74.8]	< 0.001
LVEF, %	39.6 [39.6, 52.2]	40.7 [31.4, 51.4]	55.0 [40.0, 62.0]	< 0.001
Condition, n (%)				
STEMI	140 (52)	27 (53)	113 (52)	1.00
Non-STEMI	128 (48)	24 (47)	104 (48)	1.00
GRACE risk score	130.0 [107.3, 157.0]	166 [132, 188]	124 [104, 148]	< 0.001
GNRI	102.4 [94.6, 109.5]	86.4 [77.2, 89.6]	105.1 [99.5, 111.9]	< 0.001

Values are shown as median with interquartile range

ACEI, angiotensin-converting enzyme inhibitor; ARB, angiotensin II receptor blocker; BMI, body mass index; eGFR, estimated glomerular filtration rate; FPG, fasting plasma glucose; GNRI, Geriatric Nutritional Risk Index; GRACE, Global Registry of Acute Coronary Events; HDL-C, high-density lipoprotein cholesterol; LVEF, left ventricular ejection fraction; LDL-C, low-density lipoprotein cholesterol; STEMI, ST-elevation myocardial infarction

### Mortality after PCI in the malnourished and non-malnourished groups

**Fig. 1:**
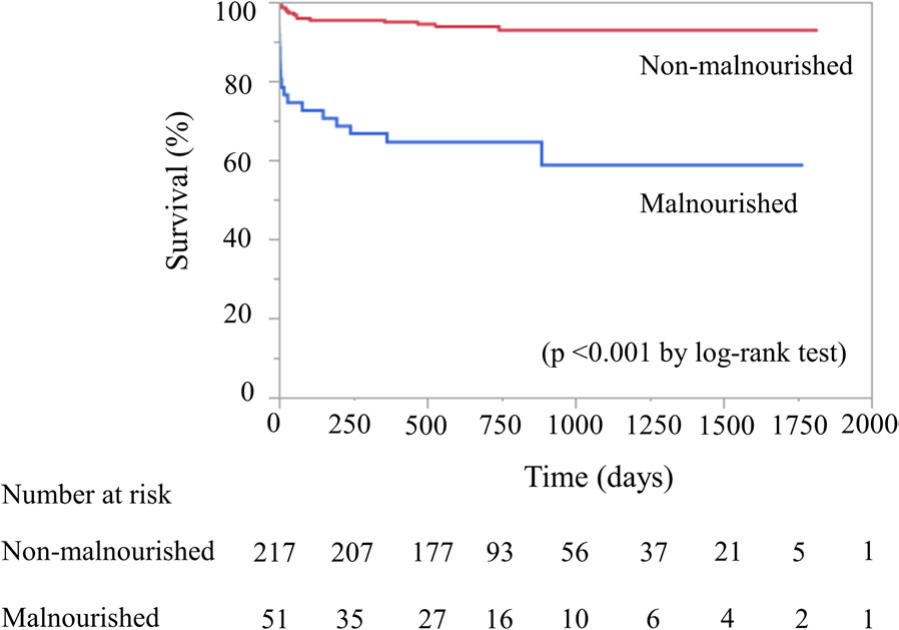
Kaplan–Meier survival curves for all-cause death after percutaneous coronary intervention

Median duration of follow-up was 698 days (IQR, 503–957 days), with a maximum follow-up of 1819 days. Thirty-three patients (12%) died after PCI (Table 2), with 24 cardiac deaths (heart failure, n = 17; cardiac rupture, n = 4; ventricular fibrillation, n = 3) and 9 non-cardiac deaths (cerebral infarction, n = 2; sepsis, n = 3; cancer, n = 4). The incidences of all-cause and cardiac deaths were significantly higher in the malnourished group than in non-malnourished group within 1 month after PCI (p < 0.001 each), beyond 1 month after PCI (p = 0.017 and p = 0.002), and for the full follow-up period after PCI (p < 0.001 each) (Table 2). Kaplan–Meier analysis showed a significantly lower survival rate in the malnourished than in the non-malnourished group (p < 0.001) (Fig. 1).

**Table 2:** Cumulative deaths after PCI based on nutritional status

	Overall	Malnourished group	Non-malnourished group	P value
	(n = 268)	(n = 51)	(n = 217)	
Within 1 month after PCI				
All-cause death	17 (6%)	12 (24%)	5 (2%)	< 0.001
Cardiac death	15 (6%)	11 (22%)	4 (2%)	< 0.001
Non-cardiac death	2 (1%)	1 (4%)	1 (1%)	0.32
Beyond 1 month after PCI				
All-cause death	16 (6%)	8 (16%)	8 (4%)	0.017
Cardiac death	9 (3%)	6 (12%)	3 (1%)	0.002
Non-cardiac death	7 (3%)	2 (4%)	5 (2%)	0.62
Full follow-up period after PCI				
All-cause death	33 (12%)	20 (39%)	13 (6%)	< 0.001
Cardiac death	24 (9%)	17 (33%)	7 (3%)	< 0.001
Non-cardiac death	9 (3%)	3 (6%)	6 (3%)	0.38

PCI, percutaneous coronary intervention

### Influence of baseline characteristics on all-cause deaths after PCI

Univariate Cox proportional hazards regression analysis revealed that both GRACE risk score and malnutrition were positively associated with all-cause death within 1 month after PCI (p < 0.001 and p = 0.001), beyond 1 month after PCI (p < 0.001 and p = 0.003), and full follow-up period after PCI (p < 0.001 and p < 0.001) and LVEF negatively related to all-cause death within one month after PCI (p = 0.025) and full follow-up period (p < 0.001) (Table 3). The multivariate cox proportional hazards regression model revealed that there was a strong association between malnutrition and all-cause death within 1 month after PCI (model 3, HR: 7.04, 95%CI: 2.30–21.51, p < 0.001), beyond 1 month after PCI (model 3, HR: 3.10, 95%CI: 1.70–8.96, p = 0.037), and full follow-up period after PCI (model 3, HR: 5.64, 95%CI: 1.97–16.13, p = 0.001), even after adjusting for the relevant factors, as previously mentioned (Table 4). BMI and albumin were used to calculate GNRI. As a result, these factors were not incorporated into the multivariate analysis.

**Table 3:** Univariate Cox proportional hazards regression analysis for all-cause death

	Within 1 month after PCI		Beyond 1 month after PCI		Full follow-up period after PCI	
	HR (95%CI)	P value	HR (95%CI)	P value	HR (95%CI)	P value
Age	1.04 (0.98–1.11)	0.20	1.03 (0.99–1.08)	0.170	1.04 (0.99–1.08)	0.08
Sex: male	2.08 (0.60–7.25)	0.21	0.75 (0.27–2.07)	0.58	1.19 (0.56–2.57)	0.64
BMI	0.83 (0.68–0.99)	0.048	0.88 (0.75–1.01)	0.068	0.85 (0.74–0.96)	0.011
Hypertension	1.77 (0.47–6.58)	0.40	1.68 (0.61–4.64)	0.32	1.63 (0.65–4.06)	0.29
Diabetes mellitus	1.56 (0.42–5.82)	0.51	1.26 (0.47–3.37)	0.63	1.73 (0.69–4.33)	0.24
Dyslipidaemia	0.62 (0.16–2.31)	0.48	0.85 (0.31–2.33)	0.75	0.68 (0.27–1.69)	0.41
Current smoker	1.67 (0.72–4.88)	0.57	1.10 (0.36–3.44)	0.86	1.46 (0.42–5.02)	0.55
Haemodialysis	1.32 (0.17–10.56)	0.79	1.63 (0.17–10.56)	0.79	3.01 (0.40–22.51)	0.28
Aspirin	0.50 (0.11–2.45)	0.40	0.88 (0.31–2.54)	0.82	0.80 (0.31–2.12)	0.66
Thienopyridines	0.89 (0.26–3.11)	0.85	0.25 (0.03–1.92)	0.104	0.55 (0.19–1.56)	0.23
Dual antiplatelet therapy	0.92 (0.11–7.32)	0.93	0.44 (0.05–3.35)	0.37	0.83 (0.19–3.60)	0.81
Oral anticoagulation	1.47 (0.18–11.75)	0.72	2.04 (0.46–8.98)	0.40	2.18 (0.63–7.49)	0.22
Calcium-channel blocker	0.96 (0.24–3.86)	0.96	0.80 (0.27–2.28)	0.66	0.88 (0.33–2.31)	0.79
ACEI	1.52 (0.33–6.28)	0.56	2.43 (0.55–10.71)	0.294	1.56 (0.36–6.76)	0.55
ARB	0.33 (0.04–2.66)	0.29	0.89 (0.31–2.58)	0.84	0.47 (0.14–1.63)	0.24
β-blocker	0.49 (0.10–2.37)	0.38	0.39 (0.05–2.97)	0.30	0.82 (0.19–3.57)	0.80
Statin	0.46 (0.10–2.28)	0.35	0.91 (0.33–2.52)	0.87	0.75 (0.28–1.96)	0.56
LDL-C	0.98 (0.96–1.04)	0.12	0.99 (0.97–1.01)	0.28	0.98 (0.96–0.99)	0.062
HDL-C	0.98 (0.92–1.03)	0.43	0.98 (0.93–1.03)	0.46	0.98 (0.94–1.03)	0.52
Triglyceride	0.98 (0.96–1.00)	0.060	0.99 (0.98–1.00)	0.44	0.99 (0.98–1.01)	0.054
Albumin	0.22 (0.09–0.53)	< 0.001	0.32 (0.16–0.67)	0.003	0.21 (0.11–0.38)	< 0.001
FPG	0.98 (0.97–1.00)	0.20	1.00 (0.99–1.01)	0.45	1.00 (0.99–1.00)	0.99
eGFR	0.99 (0.96–1.01)	0.25	0.99 (0.97–1.01)	0.30	0.98 (0.97–1.00)	0.085
LVEF	0.94 (0.90–0.99)	0.025	0.97 (0.94–1.01)	0.112	0.93 (0.91–0.97)	< 0.001
GRACE risk score	1.03 (1.01–1.04)	< 0.001	1.02 (1.01–1.03)	< 0.001	1.03 (1.02–1.04)	< 0.001
Malnutrition (GNRI < 92.0)	10.30 (2.57–41.21)	0.001	4.59 (1.71–12.32)	0.003	9.73 (3.83–24.75)	< 0.001

CI, confidence interval; HR, hazard ratio. Other abbreviations are as in Tables 1 and 2

### Predictive values of GRACE risk score and GNRI for mortality

**Fig. 2:**
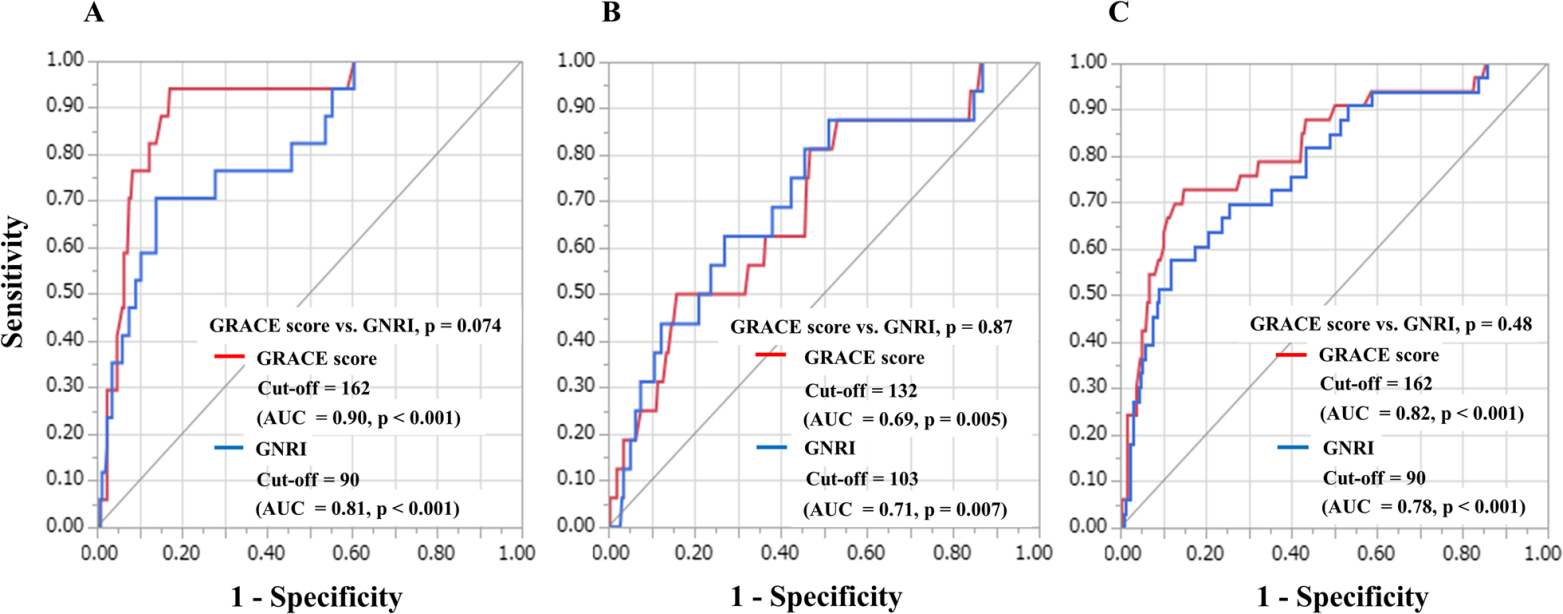
Receiver-operating characteristic curves for predicting all-cause death. **A** within 1 month after PCI, **B** beyond 1 month after PCI, **C** during the full follow-up period after PCI. AUC, area under the curve; GNRI, Geriatric Nutritional Risk Index; GRACE, Global Registry of Acute Coronary Events; PCI, percutaneous coronary intervention

**Fig. 3:**
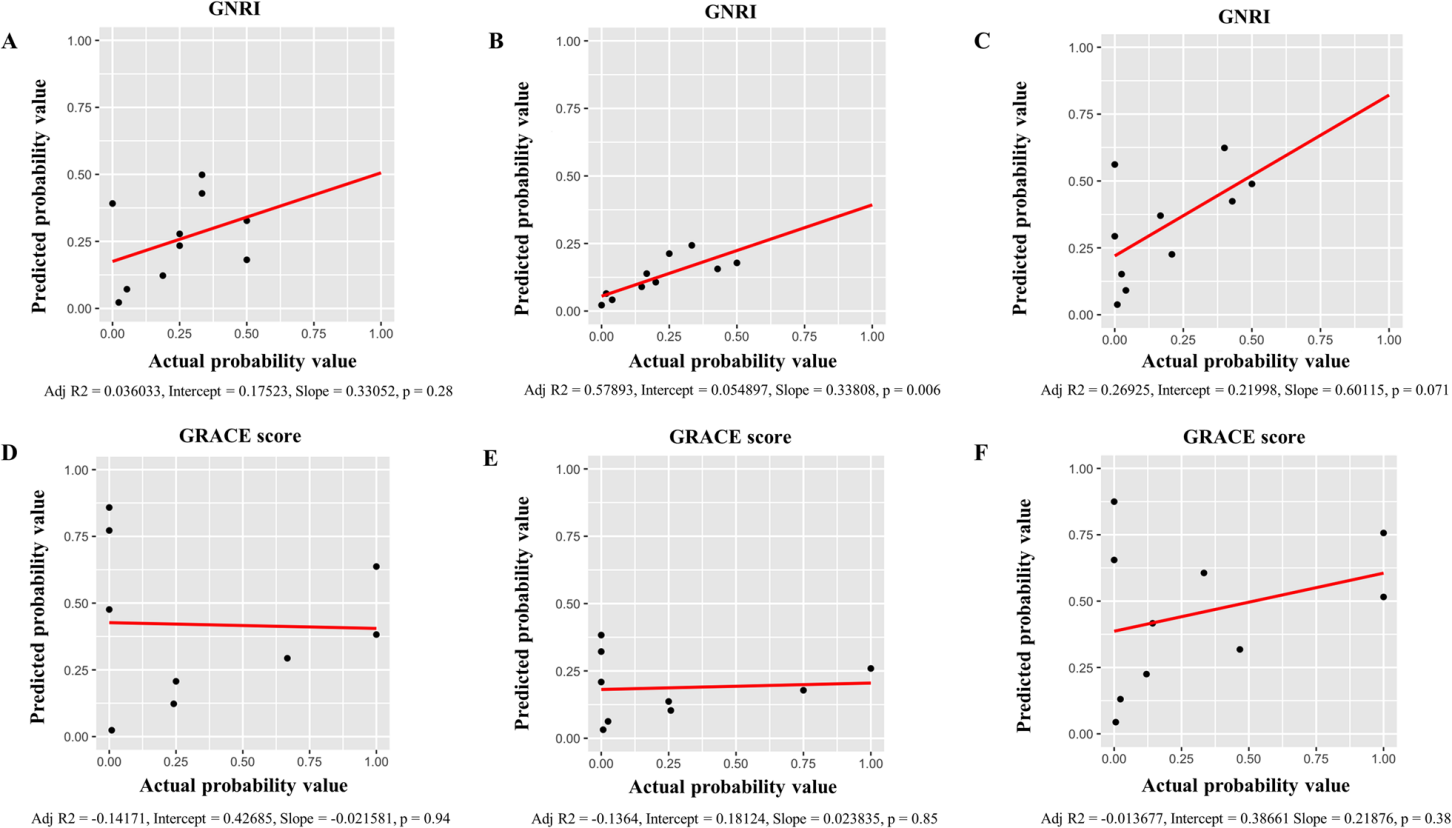
Calibration plots comparing predicted and actual mortality of GNRI and GRACE score models. **A**, **D** within 1 month after PCI, **B**, **E** beyond 1 month after PCI, **C**, **F** during the full follow-up period after PCI. GNRI, Geriatric Nutritional Risk Index; GRACE, Global Registry of Acute Coronary Events; PCI, percutaneous coronary intervention

These results show that malnutrition, as assessed by GNRI, offers a potent independent risk factor of all-cause death after PCI. In the present study, GRACE risk score was also associated with all-cause death after PCI (Table 4). We therefore analysed ROC curves to evaluate the discriminatory capacities of GRACE risk score and GNRI for predicting all-cause death after PCI (Fig. 2). ROC cut-offs for GRACE risk score and GNRI in all-cause death were 162 and 90 (AUC = 0.90, p < 0.001 and AUC = 0.81, p < 0.001) within 1 month after PCI (Fig. 2A), 132 and 103 (AUC = 0.69, p = 0.005 and AUC = 0.71, p = 0.007) beyond 1 month after PCI (Fig. 2B), and 162 and 90 (AUC = 0.82, p < 0.001 and AUC = 0.78, p < 0.001) for the full follow-up period after PCI (Fig. 2C). Comparisons of ROC curves for GRACE risk score and GNRI for predicting all-cause death after PCI showed no significant difference in AUCs between GRACE risk score and GNRI, irrespective of the follow-up period (Fig. 2). The goodness of fit of logistic analysis for GRACE risk score and GNRI for predicting all-cause death after PCI were analysed by Hosmer and Lemeshow test (GRACE score; within 1 month: p = 0.71, beyond 1 month: p = 0.31, the full follow-up period: p = 0.38 and GNRI; within 1 month: p = 0.43, beyond 1 month: p = 0.50, the full follow-up period: p = 0.45). In addition, Calibration plots comparing actual and predicted mortality were shown in Fig. 3, and GNRI had a significant correlation only beyond 1 month after PCI (Fig. 3B). Furthermore, we conducted a test for interaction of malnutrition and GRACE risk score. As a result, comparison of p-value for interaction of malnutrition and GRACE risk score for all-cause death within 1 month after PCI, beyond 1 month after PCI, and the full follow-up period after PCI were p = 0.62, p = 0.64 and p = 0.38, respectively.

**Table 4:** Predictive values of malnutrition for all-cause death as determined by multivariate Cox proportional hazards regression analysis modelling

	Model 1			Model 2			Model 3		
	HR	95%CI	P value	HR	95%CI	P value	HR	95%CI	P value
Within 1 month after PCI									
Age	0.98	0.94–1.03	0.42	1.02	0.95–1.10	0.95	0.96	0.92–1.01	0.07
LVEF	–	–	–	0.93	0.89–0.97	< 0.001	–	–	–
GRACE risk score	–	–	–	–	–	–	1.03	1.01–1.04	< 0.001
Malnutrition	13.08	4.41–38.81	< 0.001	6.74	2.13–21.33	0.001	7.04	2.30–21.51	< 0.001
Beyond 1 month after PCI									
Age	1.01	0.97–1.06	0.55	1.02	0.97–1.07	0.44	0.99	0.94–1.04	0.70
LVEF	–	–	–	0.98	0.94–1.01	0.216	–	–	–
GRACE risk score	–	–	–	–	–	–	1.02	1.01–1.03	0.002
Malnutrition	4.11	1.44–11.74	0.008	3.47	1.15–10.39	0.032	3.10	1.70–8.96	0.037
Full follow-up period after PCI									
Age	0.99	0.97–1.03	0.85	1.01	0.97–1.04	0.71	0.98	0.94–1.03	0.51
LVEF	–	–	–	0.95	0.93–0.98	< 0.001	–	–	–
GRACE risk score	–	–	–	–	–	–	1.02	1.01–1.03	0.002
Malnutrition	7.38	3.55–15.32	< 0.001	5.82	2.05–16.50	< 0.001	5.64	1.97–16.13	0.001

Model 1, adjusted for age; Model 2, adjusted for variables in Model 1 plus LVEF; Model 3, adjusted for variables in Model 1 plus GRACE risk score. CI, confidence interval; HR, hazard ratio. Other abbreviations are as in Tables 1 and 2

## Discussion

The main findings of the present study were as follows: (1) malnutrition offered an independent risk factor for all-cause death within 1 month after PCI, beyond 1 month after PCI, and during the full follow-up period in AMI patients undergoing PCI; (2) in addition to GRACE risk score, nutritional assessment by GNRI may separately be able to predict the mortality in AMI patients especially in long-term mortality.

Nutritional status is an important factor that affects the prognosis of various diseases [14, 15]. Previous studies have demonstrated that malnutrition as evaluated by BMI or serum albumin level is associated with cardiovascular events in symptomatic heart failure patients [16–19]. Furthermore, an association has also been reported between low serum albumin level and poor prognosis among coronary artery disease patients [20, 21]. BMI and serum albumin are important indicators for evaluating the risk and prognosis of cardiovascular disease. However, BMI is influenced by several non-nutritional factors, including fluid status, renal dysfunction, and inflammation [16, 22, 23]. Serum albumin is also influenced by inflammation and liver and renal functions. BMI or albumin alone may thus be insufficient for assessing nutritional status. GNRI, as an index combining BMI and albumin, has recently seen wide use as an index for evaluating nutritional status, and has also been reported as a useful parameter for determining prognosis in patients with haemodialysis, chronic heart failure, coronary artery disease and critical limb ischemia [11, 12, 24]. However, few reports have examined the relationships between GNRI and prognosis among AMI patients after PCI. The present study demonstrated that a low GNRI represented an independent risk factor for all-cause death in both the short- and long-term periods after PCI in AMI patients.

Malnutrition leads to sarcopenia, and represents a powerful predictor of morbidity and mortality [25]. Based on the accumulated evidence, frailty and sarcopenia are important factors affecting the prognosis of patients with cardiovascular disease [7]. Abnormalities in body composition such as low muscle mass are powerful predictors of morbidity and mortality, particularly in clinical settings where the disease or illness itself can lead to this condition [25]. In AMI patients, long-term intensive care is often required due to the unstable circulation caused by myocardial damage, inflammation and various complications that lead to reduced skeletal muscle, which can result in the development of sarcopenia. Furthermore, heart failure (HF) status including chronic systemic inflammation and autonomic nervous disorder in patients with after AMI affects the on changes in skeletal muscle, and inflammatory mediators released into the circulation accompanied with the progression of HF further activate systemic inflammation and promote muscle atrophy [26, 27]. Even if without heart failure, a decrease in muscle mass can be triggered along with a decrease in activity after the onset of AMI. As a result, sarcopenia often occur after the onset of AMI. Therefore, patients with malnutrition at the time of hospital admission are more susceptible to progression of sarcopenia. Malnutrition consists of a severe state that leads to a reduction in protein reserves and caloric storage, thereby weakening immune defences. Although several nutrients, particularly protein and amino acids, are required to maintain skeletal muscle weight and prevent sarcopenia [28], a way to immediately improve malnutrition has not been identified. Consequently, malnourished patients are considered to be at a distinct disadvantage during hospitalization and after discharge. The malnutrition examined in the present study would thus seem likely to be associated with poor short- and long-term prognosis among AMI patients after PCI.

Prompt assessment of mortality risk in patients with AMI is necessary for determining the optimal medical management of these patients. The GRACE risk score is widely used worldwide as a scoring system for stratifying patients diagnosed with AMI, as a means of estimating mortality [2, 3]. While GRACE risk score is primarily assessed based on circulatory status such as heart rate, systolic blood pressure and Killip class [2], the GNRI is assessed based on nutritional status such as BMI and albumin level [10]. Although the formulas for GRACE risk score and GNRI are quite different, no significant differences were seen in the predictions of all-cause death during the full follow-up period after PCI when assessed using GNRI and GRACE risk score. The GRACE risk score was originally based on an assessment of in-hospital mortality. We thus investigated all-cause deaths in the short term (within 1 month after PCI) and long term (beyond 1 month after PCI), but predictions of all-cause deaths derived from GRACE risk score and GNRI did not differ significantly. Furthermore, we conducted a test for interaction of malnutrition and GRACE risk score. These results revealed that there were no significant interactions between malnutrition and GRACE risk score in our study. The present results suggest the utility and importance of assessing nutritional status when predicting all-cause mortality after PCI, separate from the circulation status.

As mentioned above, GRACE risk score and GNRI may separately have predictive value for all-cause death after PCI. In addition to the GRACE score, which takes into account the effects of unstable circulatory dynamics associated with coronary artery occlusion, it can be inferred that assessment of nutritional status using GNRI is important for predicting all-cause mortality especially in the long term beyond 1 month.

Several limitations should be considered in regard to this present study. First, the present study used a retrospective study that contained a relatively small number of patients. Second, although we were able to demonstrate an independent association between all-cause death in AMI patients who underwent PCI and were malnourished (low GNRI, which influences frailty and sarcopenia), we were unable to investigate the relationship between other frailty/sarcopenia factors, such as muscular strength and all-cause death. Third, even though GNRI was originally established as an index for patients ≥ 65 years old, many published papers have included patients < 65 years [29]. The present study also included patients < 65 years old. However, we did find that malnutrition (low GNRI) was significantly associated with all-cause death.

## Conclusions

GNRI may have a potential for predicting the mortality in AMI patients especially in beyond 1 month after PCI, separate from GRACE risk score. Assessment of nutritional status may help stratify the risk of AMI mortality.

## Data Availability

The datasets used and/or analyzed during the current study are available from the corresponding author on reasonable request.

## Abbreviations

ACEI: Angiotensin-converting enzyme inhibitor
AMI: Acute myocardial infarction
ARB: Angiotensin II receptor blocker
BMI: Body mass index
CI: Confidence interval
eGFR: Estimated glomerular filtration rate
FPG: Fasting plasma glucose
GNRI: Geriatric Nutritional Risk Index
GRACE: Global Registry of Acute Coronary Events
HDL-C: High-density lipoprotein cholesterol
HR: Hazard ratio
IQR: Interquartile range
LVEF: Left ventricular ejection fraction
LDL-C: Low-density lipoprotein cholesterol
PCI: Percutaneous coronary intervention
STEMI: ST-elevation myocardial infarction
